# Protein quality control and antigen presentation in the development of hepatitis B-related hepatocellular carcinoma

**DOI:** 10.3389/fphar.2026.1786614

**Published:** 2026-04-10

**Authors:** Lina An, Yidi Han, Xiaohui Sun, Lili Wang, Lei Guo

**Affiliations:** 1 Department of Integrated Traditional Chinese and Western Medicine, Qingdao Public Health Clinical Center (Qingdao Sixth People’s Hospital), Qingdao, Shandong, China; 2 Department of Interventional Medicine, Qingdao Public Health Clinical Center (Qingdao Sixth People’s Hospital), Qingdao, Shandong, China; 3 Department of Research and Education, Qingdao Public Health Clinical Center (Qingdao Sixth People’s Hospital), Qingdao, Shandong, China; 4 Department of Liver Oncology, Qingdao Public Health Clinical Center (Qingdao Sixth People’s Hospital), Qingdao, Shandong, China

**Keywords:** antigen presentation, hepatitis B virus, hepatocellular carcinoma, protein quality control, unfolded protein response

## Abstract

Chronic hepatitis B virus (HBV) infection remains a leading cause of hepatocellular carcinoma (HCC) worldwide. Although viral integration, chronic inflammation, and immune-mediated injury are established contributors to carcinogenesis, emerging evidence indicates that hepatocellular protein quality control (PQC) pathways and antigen presentation mechanisms play central mechanistic roles in shaping the cellular and immunological environment in which HBV-related HCC develops. HBV imposes an extraordinary folding and degradation burden on hepatocytes through massive production of viral proteins and persistent engagement of the endoplasmic reticulum (ER), cytosolic degradation pathways, and autophagy machinery. Dysregulation of the unfolded protein response (UPR), ER-associated degradation (ERAD), the ubiquitin-proteasome system (UPS), and autophagy enables hepatocytes to tolerate sustained proteotoxic stress while accumulating DNA damage, metabolic alterations, and oncogenic mutations. Simultaneously, HBV disrupts antigen processing and presentation by impairing immunoproteasome function, inhibiting peptide transport and loading, and reducing MHC class I expression. The convergence of PQC dysfunction and antigen presentation impairment is proposed to promote persistent immune evasion, clonal expansion of damaged hepatocytes, thereby increasing the likelihood of malignant transformation. This review integrates recent mechanistic insights into PQC pathways, HBV-host interactions, antigen presentation defects, and their combined contributions to the evolution of HBV-related HCC.

## Introduction

Hepatocellular carcinoma (HCC) is the most common primary liver cancer and a major global health burden (Sheena et al., 2022). Chronic infection with hepatitis B virus (HBV) accounts for the majority of HCC cases in East Asia and sub-Saharan Africa and remains a critical etiological factor even in regions with widespread antiviral therapy (El-Serag HB, 2012). While immune-mediated liver injury, viral DNA integration, and chronic inflammation have long been recognized as fundamental components of HBV-driven carcinogenesis, accumulating evidence indicates that disruption of hepatocyte protein homeostasis and antigen presentation plays a central, mechanistically coherent role in the transition from chronic hepatitis to malignancy (Hao et al., 2025).

Hepatocytes infected with HBV sustain an exceptional protein synthesis burden. HBV produces high levels of surface antigen (HBsAg) and secretes vast quantities of non-infectious subviral particles, often reaching concentrations orders of magnitude greater than infectious virions (Mohebbi et al., 2018). The folding and assembly of these membrane proteins require intense engagement of endoplasmic reticulum (ER) chaperones, glycosylation machinery, and protein quality control (PQC) pathways (Zhang Y-Z. et al., 2022). HBV core antigen, e antigen, polymerase, and the regulatory protein HBx further increase cytosolic and ER folding loads. This continuous influx of viral proteins engages the unfolded protein response (UPR), ER-associated degradation (ERAD), the ubiquitin-proteasome system (UPS), and autophagy. These pathways initially function to protect the cell from proteotoxicity; however, persistent stimulation by viral proteins causes profound and long-lasting alterations in hepatocyte proteostasis (Lazar et al., 2014).

Chronic activation of the UPR is particularly relevant to carcinogenesis. HBV-induced ER stress elicits sustained activation of IRE1α, PERK, and ATF6 signaling, pathways that modulate inflammatory cytokine production, lipid synthesis, redox homeostasis, and cell survival mechanisms (Wu et al., 2021). During prolonged infection, these adaptive responses become maladaptive, promoting oxidative stress, enhancing tolerance of DNA damage, and reshaping hepatocyte metabolism toward oncogenic programs. Viral protein accumulation also saturates ERAD capacity and overwhelms the UPS, further amplifying proteotoxic stress and contributing to mitochondrial dysfunction, oxidative injury, and genomic instability, all of which are hallmarks of early carcinogenesis (Wei and Fang, 2021).

Meanwhile, HBV subverts the PQC machinery for its own replication. HBx interacts with the DDB1-CUL4 E3 ligase complex to degrade host restriction factors, including the SMC5/6 complex, thereby enabling viral transcription from covalently closed circular DNA (cccDNA) (Murphy et al., 2016; Sekiba et al., 2022). This hijacking of UPS machinery alters the stability of key regulators of DNA repair, apoptosis, and cell cycle progression. Simultaneously, HBV reprograms autophagy by promoting autophagosome formation but inhibiting fusion with lysosomes, creating a partially active autophagy state that supports viral nucleocapsid assembly while compromising cellular turnover of damaged organelles (Wang J. et al., 2019).

A second major consequence of HBV-driven PQC disruption is impairment of antigen presentation. The proteasome and immunoproteasome are responsible for generating peptides that bind MHC class I molecules, yet HBV suppresses immunoproteasome induction by antagonizing interferon signaling (Robek et al., 2007). Viral proteins inhibit TAP-mediated peptide transport, destabilize tapasin and other components of the peptide-loading complex, and impair folding of MHC-I heavy chains under chronic ER stress (Montali et al., 2022). These combined defects dramatically reduce the quantity and quality of viral peptides displayed on the hepatocyte surface, allowing HBV-infected cells to escape cytotoxic T-cell recognition. This immunological invisibility enables survival of hepatocytes harboring oncogenic lesions and contributes directly to the emergence of pre-neoplastic foci (Zheng et al., 2023).

Thus, PQC dysregulation and antigen presentation impairment are deeply intertwined drivers of HBV-related liver cancer. PQC pathways determine how hepatocytes respond to the toxic burden of viral proteins, while antigen presentation dictates the ability of the immune system to eliminate damaged or transformed hepatocytes. When both systems are persistently perturbed, as may occur during chronic HBV infection, conditions may emerge that favor malignant transformation through sustained survival of genomically unstable cells, persistent inflammatory and metabolic stress, and long-term immune evasion.

This review synthesizes mechanistic advances from molecular virology, cell biology, immunology, and cancer research to build an integrated model of how PQC pathways and antigen presentation shape the evolution of HBV-related HCC. We first examine the structure and regulation of PQC systems in hepatocytes, then discuss how HBV manipulates each pathway. We next analyze how chronic ER stress, UPS overload, and autophagy disruption contribute to hepatocyte transformation. Finally, we describe how antigen presentation failures reinforce immune escape and highlight emerging therapeutic strategies targeting PQC-immune crosstalk to prevent or treat HBV-associated HCC. In this review, we propose that the crosstalk between hepatocellular protein quality control (PQC) pathways and antigen presentation does not merely accompany HBV-related hepatocarcinogenesis but constitutes a mechanistic axis that links chronic viral proteotoxic stress to immune escape and clonal selection. We position PQC-antigen presentation dysfunction as a unifying intermediate layer between viral persistence and malignant evolution, integrating intracellular stress adaptation with impaired immunological visibility.

## Overview of protein quality control in hepatocytes

Hepatocytes operate under high biosynthetic load, synthesizing plasma proteins, lipoproteins, metabolic enzymes, and detoxifying complexes. Their survival depends on a finely tuned protein quality control (PQC) network comprising the endoplasmic reticulum (ER) protein-folding machinery, ER-associated degradation (ERAD), the ubiquitin-proteasome system (UPS), and autophagy (Hetz et al., 2011; Walter and Ron, 2011). These pathways collectively maintain proteostasis by ensuring that newly synthesized proteins fold correctly and that damaged or misfolded proteins are efficiently removed (Hetz and Papa, 2018).

The ER is the principal folding compartment in hepatocytes. As secretory and membrane proteins enter the ER, they engage with chaperones such as BiP/GRP78, GRP94, calnexin, and calreticulin, which stabilize nascent polypeptides, facilitate disulfide bond formation, and determine whether proteins progress to the Golgi or undergo degradation (Nakatsukasa et al., 2008). Misfolded proteins are recognized by lectin-like sensors and routed to ERAD, where they are retrotranslocated to the cytosol, ubiquitinated by ligases such as the SEL1L-HRD1 complex, and degraded by the proteasome (Sun and Brodsky, 2019). ERAD plays a central role in maintaining the secretory capacity of hepatocytes, and defects in ERAD result in ER distension, steatosis, and inflammation—phenotypes that closely resemble early pathological changes in chronic hepatitis B (Liu et al., 2020).

Persistent ER stress activates the unfolded protein response (UPR), a transcriptional and translational adaptation mediated by three ER transmembrane sensors: IRE1α, PERK, and ATF6 (Hetz et al., 2020). Activated IRE1α splices XBP1 mRNA to produce XBP1s, a transcription factor that enhances ER folding capacity, lipid synthesis, and ERAD efficiency (Barton et al., 2025; Sha et al., 2009). PERK phosphorylates eIF2α, reducing global translation while promoting selective translation of ATF4, which governs antioxidant responses, amino acid metabolism, and apoptosis (Rozpedek et al., 2016). ATF6 traffics to the Golgi for proteolytic activation and induces genes that expand ER volume and increase chaperone levels (Shen et al., 2002). Short-lived UPR activation restores homeostasis; however, chronic or dysregulated UPR signaling contributes to oxidative stress, metabolic remodeling, and survival of damaged cells (Maity et al., 2016; Fu et al., 2025).

UPS complements these pathways by degrading misfolded cytosolic proteins, regulatory factors, and damaged enzymes. The 26S proteasome also produces peptides for loading onto MHC class I molecules, linking PQC to antigen presentation (Cascio et al., 2001). Immunoproteasomes, induced by interferons, generate peptides optimized for MHC-I presentation and enhance antiviral immunity (Dimasuay et al., 2022). Autophagy, the lysosomal degradation pathway, eliminates long-lived proteins, aggregates, and dysfunctional organelles (Mizushima and Komatsu, 2011). Selective autophagy subtypes, such as mitophagy and ER-phagy, maintain organelle health, while lipophagy regulates hepatic lipid metabolism (Reggiori and Molinari, 2022; Minami et al., 2023). Together, these PQC pathways maintain hepatocyte function under physiological and stress conditions.

Disruption of any component of PQC results in compensatory activation of others. When ERAD or the UPS is overloaded, autophagy is upregulated to degrade accumulated substrates (Pla-Prats and Thomä, 2022). Conversely, impaired autophagy increases the burden on the proteasome (Korolchuk et al., 2009). Hepatitis B virus exploits this interconnected network, reshaping PQC in ways that support viral replication and progressively distort hepatocyte physiology, ultimately facilitating carcinogenesis (Wang X. et al., 2022; Han et al., 2025).

## HBV biology relevant to PQC and antigen processing

HBV is a small, enveloped DNA virus with a compact 3.2-kb genome encoding four overlapping open reading frames that generate surface (S), core (C), polymerase (P), and regulatory X (HBx) proteins. After entry into hepatocytes, HBV delivers relaxed circular DNA to the nucleus, where it is converted to covalently closed circular DNA (cccDNA)-the stable viral minichromosome. Transcription from cccDNA produces viral proteins and pgRNA, whose translation and replication impose significant proteostatic demands on the host cell (Liang, 2009; Caligiuri et al., 2016).

Among HBV proteins, HBsAg is produced in enormous excess, especially as non-infectious subviral particles that can reach concentrations millions of times higher than virions. Large (pre-S1/pre-S2), middle, and small HBsAg isoforms undergo extensive glycosylation and membrane insertion, making them particularly demanding on the ER folding machinery (Chai et al., 2008). Accumulation of misfolded HBs proteins triggers ER stress, and pre-S deletion mutants can aggregate within the ER, producing distinct cytopathic features associated with pre-neoplastic lesions (Hu et al., 2022).

HBcAg assembles into capsids in the cytosol, where stoichiometric imbalances between core protein, polymerase, and pgRNA increase proteotoxic load (Melegari et al., 2005; Tan et al., 2015). HBeAg, a secretory protein, contributes to ER stress through its transit and folding requirements (Uchida et al., 2023). Together, the HBV proteome represents a persistent challenge to PQC systems. HBx is the viral protein most directly involved in manipulating host PQC and antigen presentation. HBx binds to DDB1, a substrate adaptor of the CRL4 E3 ubiquitin ligase complex, redirecting substrate specificity to favor viral replication (Murphy et al., 2016). The best-characterized target is the SMC5/6 complex, a restriction factor that represses cccDNA transcription; its HBx-mediated degradation is essential for viral gene expression (Sekiba et al., 2022). HBx also destabilizes regulators of DNA repair, apoptosis, chromosomal stability, and mitotic progression, promoting cellular environments conducive to oncogenic evolution (Hung et al., 2024).

HBV additionally exploits autophagy by stimulating autophagosome formation through HBx-mediated activation of Beclin-1 and PI3KC3 complexes (Wang J. et al., 2019; Abdoli et al., 2019). However, the virus inhibits autophagosome-lysosome fusion, preserving autophagosomes as replication platforms while impairing the cell’s ability to eliminate damaged organelles. This incomplete autophagy promotes mitochondrial dysfunction, oxidative stress, and accumulation of toxic aggregates, all of which contribute to malignant transformation (Lin et al., 2019; Schollmeier et al., 2024).

**FIGURE 1:**
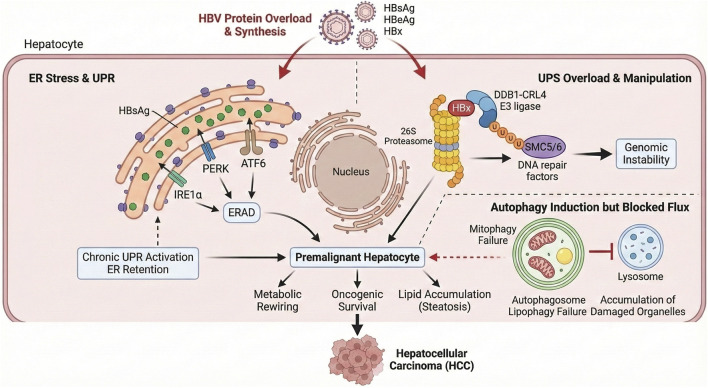
Mechanisms of HBV-induced Carcinogenesis via PQC Failure. The diagram illustrates how HBV proteins (HBsAg, HBx) disrupt cellular homeostasis through three axes (1): Inducing chronic ER stress and the Unfolded Protein Response (UPR) (2); Hijacking the Ubiquitin-Proteasome System (UPS) via DDB1-CRL4 to degrade DNA repair factors (SMC5/6); and (3) Blocking autophagic flux, leading to the accumulation of damaged mitochondria and lipids. Together, these maladaptive responses drive genomic instability and metabolic rewiring, leading to HCC.

By simultaneously increasing folding and degradation demands, subverting UPS function, altering autophagy flux, and suppressing antigen processing, HBV profoundly alters hepatocyte homeostasis and enables persistent infection and carcinogenesis. (Figure 1).

## HBV-induced ER stress and the unfolded protein response in liver cancer development

The continuous synthesis and folding of HBV proteins induce chronic ER stress, a defining feature of HBV-infected hepatocytes. Although the UPR initially mitigates damage by enhancing folding capacity, expanding the ER, and reducing global translation, chronic activation reprograms hepatocyte biology in ways that foster oncogenesis.

HBsAg accumulation is the principal trigger of ER stress. Large HBs variants and pre-S mutants form aggregates in the ER lumen and are inefficiently trafficked through the secretory pathway. Their retention induces ER membrane proliferation, chaperone induction, and architectural distortion of the ER network (Wang et al., 2006; Liang et al., 2021). Histological studies demonstrate that pre-S mutant-containing “ground-glass hepatocytes” frequently appear adjacent to early HCC lesions, reinforcing the connection between ER stress and carcinogenesis (Su et al., 2008; Mathai et al., 2013).

The IRE1α-XBP1 branch is persistently activated in HBV-infected hepatocytes (Hu et al., 2022). XBP1s enhances ER biogenesis, lipid synthesis, and ERAD gene expression, helping hepatocytes withstand the folding burden (Lee et al., 2008). However, chronic IRE1α signaling activates JNK pathways that promote inflammatory cytokine production, including IL-6 and TNF-α (Wu et al., 2018). These cytokines sustain a pro-tumorigenic microenvironment, stimulate compensatory proliferation, and promote survival of damaged hepatocytes. Moreover, XBP1s-mediated lipogenesis contributes to steatosis, increasing susceptibility to lipotoxicity and ROS production, both of which accelerate DNA damage (Bang et al., 2019; Wang Q. et al., 2022).

The PERK-eIF2α-ATF4 pathway is similarly reprogrammed during chronic infection. PERK activation lowers translation rates, reducing ER burden but also diminishing synthesis of antigen-processing machinery. ATF4 promotes antioxidant responses and amino acid metabolism, allowing hepatocytes to tolerate high oxidative stress (Luo et al., 2018). While CHOP induction can promote apoptosis, HBV dampens CHOP-dependent pathways, skewing PERK signaling toward survival rather than death. This adaptation enables hepatocytes harboring DNA damage or chromosomal abnormalities to persist (Zhou et al., 2024).

ATF6 activation contributes to ER expansion and enhances chaperone availability. Its downstream targets also influence lipid homeostasis (Tam et al., 2018). Sustained ATF6 signaling in HBV-infected hepatocytes correlates with increased phospholipid synthesis, altered lipoprotein secretion, and metabolic remodeling that resembles patterns observed in HCC (Maiers and Malhi, 2019; Lin et al., 2025).

Together, the combined hyperactivation of IRE1α, PERK, and ATF6 generates a survival-focused UPR signature that allows hepatocytes to endure chronic proteotoxic stress. However, this altered UPR environment comes at a cost: enhanced tolerance of DNA damage, mitochondrial dysfunction, oxidative stress, dysregulated lipid metabolism, and suppression of apoptosis. Collectively, these factors may favor the survival and expansion of hepatocyte populations carrying oncogenic alterations.

ER stress also impairs antigen presentation. Chronic UPR activation reduces MHC-I folding efficiency, accelerates degradation of peptide-loading components, decreases TAP expression, and weakens immunoproteasome induction (Guttman et al., 2022). The consequence is a dramatic reduction in antigenic peptide availability and surface MHC-I expression, enabling immune evasion. Hepatocytes undergoing oncogenic transformation typically express neoantigens or altered self-antigens that could be detected by cytotoxic T cells. However, when ER stress simultaneously suppresses peptide generation, loading, and MHC-I trafficking, such emerging lesions may be less efficiently cleared by cytotoxic T cells under conditions of reduced antigen presentation (Qin et al., 2020; Dhatchinamoorthy et al., 2021).

Finally, ER stress amplifies inflammatory and fibrogenic signaling in the liver microenvironment. Persistent cytokine production recruits immune cells that produce ROS and nitric oxide, increasing DNA damage and hepatocyte turnover. Repeated cycles of injury and regeneration facilitate clonal expansion and selection of mutated hepatocytes. Therefore, ER stress functions as both an intrinsic and extrinsic driver of carcinogenesis (Kay et al., 2019; Banerjee et al., 2023).

## The ubiquitin-proteasome system and HBV-driven hepatocarcinogenesis

The ubiquitin-proteasome system (UPS) is central to hepatocyte proteostasis and antiviral immunity. Through its regulation of protein turnover, degradation of misfolded substrates, and generation of peptides for MHC class I presentation, the UPS sits at a critical interface between PQC and immune surveillance. Hepatitis B virus (HBV) exploits and disturbs this system at multiple levels, thereby facilitating both viral persistence and malignant transformation.

HBV disrupts UPS function through several mechanisms, with the viral regulatory protein HBx being a primary effector. HBx binds to the DDB1 adaptor of the CRL4 E3 ligase complex, redirecting ubiquitination activity toward host restriction factors. The most well-characterized target is the SMC5/6 complex, which normally suppresses transcription from cccDNA. HBx-mediated degradation of SMC5/6 is therefore essential for viral gene expression, but this activity also destabilizes DNA repair processes, enhances chromosomal instability, and weakens cellular responses to replication stress (Sekiba et al., 2022; Decorsière et al., 2016). HBx’s interactions with UPS machinery extend beyond CRL4; studies have shown that HBx alters the stability of p53, p27, and other cell cycle regulators, shifting hepatocytes toward survival even in the presence of genomic damage (Mukherji et al., 2007).

Proteasome overload is another key aspect of UPS dysregulation during HBV infection. The enormous quantities of viral proteins synthesized in chronically infected hepatocytes saturate proteasomal capacity. Misfolded and aggregated proteins accumulate, stimulating compensatory autophagy and activating stress response pathways (Wang J. et al., 2019). Sustained overload reduces the efficiency of peptide generation for antigen presentation and increases the production of reactive oxygen species (ROS) due to insufficient clearance of oxidized proteins. Elevated ROS levels damage DNA, lipids, and mitochondria, further driving oncogenesis (Rana et al., 2023).

Adding to this disruption, HBV subverts immunoproteasome induction. During typical viral infections, interferon-γ triggers the replacement of standard proteasome catalytic subunits with LMP2, LMP7, and MECL-1, enhancing the generation of peptides suitable for MHC-I binding (Griffin et al., 1998; Ferrington and Gregerson, 2012). HBV suppresses interferon signaling and thereby prevents immunoproteasome formation. As a result, hepatocytes generate fewer optimal viral epitopes, weakening CTL responses and promoting chronic infection (De Pasquale et al., 2021). This suppression of immunoproteasome activity is particularly harmful in the context of carcinogenesis, as it reduces the presentation of tumor-associated antigens arising from early oncogenic mutations.

UPS dysfunction also contributes to metabolic reprogramming in HBV-infected hepatocytes. Several metabolic regulators-including transcription factors controlling glycolysis, lipogenesis, and mitochondrial respiration-are UPS substrates. HBx-driven changes in E3 ligase activity alter their stability, leading to metabolic patterns similar to those seen in precancerous liver disease. UPS-mediated stabilization of β-catenin further activates the Wnt signaling pathway, a major driver of HCC (Yu et al., 2022; Ma et al., 2024).

Thus, UPS dysregulation in HBV infection shapes hepatocyte biology across multiple levels: impaired protein clearance, altered stress signaling, disturbed antigen presentation, oxidative injury, and metabolic remodeling. These changes collectively support both viral persistence and the survival of damaged hepatocytes, which together lay the groundwork for malignant transformation.

## Autophagy in HBV infection and HBV-related hepatocarcinogenesis

In hepatocytes, autophagy is essential for metabolic adaptation, detoxification, mitochondrial quality control, and maintenance of cellular homeostasis (Madrigal-Matute and Cuervo, 2016). HBV profoundly alters autophagy dynamics, inducing autophagosome formation while blocking autophagic flux. This selective manipulation enhances viral replication while promoting cell survival under conditions that would otherwise induce apoptosis, thereby contributing to hepatocarcinogenesis (Wang J. et al., 2019; Takamura et al., 2011).

Autophagy initiation begins with activation of ULK1 and phosphorylation of Beclin-1 and PI3KC3/Vps34 complexes, leading to nucleation of autophagosomal membranes. HBx directly interacts with Beclin-1 and other autophagy regulators to stimulate autophagosome formation (Sir et al., 2010). This activation benefits HBV by providing membrane-rich compartments that facilitate nucleocapsid assembly (Li et al., 2011). Autophagosomes also sequester damaged proteins and organelles, partly relieving pressure on ERAD and the UPS during viral protein overload (Bernales et al., 2006).

However, HBV inhibits lysosomal fusion, preventing autophagosomal contents from undergoing degradation. HBx disrupts SNARE complexes and lysosomal acidification, resulting in an accumulation of autophagosomes (Lin et al., 2019; Liu et al., 2014). This incomplete autophagy increases intracellular levels of damaged mitochondria, protein aggregates, and ROS. In hepatocytes, ROS act as both mutagens and signaling molecules, promoting DNA double-strand breaks, lipid peroxidation, and activation of oncogenic pathways such as NF-κB and STAT3 (Takamura et al., 2011; He et al., 2010).

Autophagy dysregulation also impacts mitochondrial quality control. Efficient mitophagy is necessary to remove dysfunctional mitochondria, which otherwise generate excessive ROS and initiate apoptosis (Twig et al., 2008). In HBV infection, impaired mitophagy creates a vicious cycle of mitochondrial injury, ROS accumulation, and genomic instability. HBx localization to the mitochondrial outer membrane further disrupts mitochondrial potential and promotes fragmentation, amplifying mitophagy demand while simultaneously inhibiting its completion (Kim et al., 2013; Yoo et al., 2019).

Metabolic rewiring is another downstream effect of autophagy disruption. Hepatocytes with impaired autophagy accumulate lipid droplets due to defective lipophagy. This lipid buildup contributes to oxidative stress and ER stress, reinforcing PQC overload (Li et al., 2018; Zhang S. et al., 2022). HBV benefits from this metabolic environment: lipids serve as key components for virion envelope synthesis and facilitate assembly of viral structures. Furthermore, autophagy inhibition shifts cellular metabolism toward glycolysis, an adaptation associated with early carcinogenesis (Gupta et al., 2006; Chen et al., 2021).

Importantly, autophagy intersects with antigen presentation pathways. Inhibition of autophagic flux reduces delivery of viral antigens to lysosomes, limiting MHC-II presentation (Nimmerjahn et al., 2003). Autophagy also supports cross-presentation and can influence peptide availability for MHC-I molecules. When autophagy is impaired, proteasomal load increases, reducing antigenic peptide quality. Thus, HBV-induced autophagy manipulation not only alters hepatocyte metabolism but also contributes directly to immune escape (Takamura et al., 2011; De Pasquale et al., 2021).

The survival advantage provided by altered autophagy enables hepatocytes with DNA damage or oncogenic mutations to persist in an environment where they would normally undergo apoptosis. Over time, this promotes clonal selection and expansion, two essential steps in the evolution of HCC.

## Antigen presentation pathways in HBV infection and immune escape

Antigen presentation is central to antiviral immunity, allowing cytotoxic T cells to recognize and eliminate infected hepatocytes. HBV subverts nearly every stage of this process, from proteasomal peptide generation to MHC-I folding, loading, and trafficking. The resulting impairment permits prolonged viral persistence and allows dysplastic hepatocytes to evade immune-mediated elimination.

A major contributor to impaired antigen presentation is the suppression of interferon signaling in infected hepatocytes. HBV reduces the induction of immunoproteasome subunits normally upregulated by interferon-γ, thereby limiting the generation of high-affinity viral peptides optimized for MHC-I loading (Cho et al., 2012). Reduced peptide quantity and quality dampen cytotoxic T-cell responses and diminish immune pressure on infected or dysplastic hepatocytes (Wang et al., 2025).

HBV infection also perturbs the peptide-loading complex indirectly through cellular stress pathways. Chronic activation of the unfolded protein response alters the activity of chaperones required for MHC-I folding, including calreticulin, ERp57, and tapasin, resulting in inefficient peptide editing and accelerated ER-associated degradation of unstable MHC-I molecules. These defects further restrict the pool of mature MHC-I-peptide complexes available for surface expression (Dick et al., 2002; Kang and Cresswell, 2002).

At the transcriptional level, HBx and stress-responsive signaling pathways have been shown to reduce expression of MHC-I heavy chains and select peptide-loading components, compounding post-translational defects. The combined outcome is a marked decrease in MHC-I surface density and antigenic diversity (Singal et al., 1996; You et al., 2022).

Together, these alterations generate a state of functional antigen presentation failure. HBV-infected hepatocytes display insufficient peptide-MHC complexes to sustain effective CD8^+^ T-cell activation, and the tolerogenic hepatic microenvironment further promotes T-cell exhaustion. In this context of weakened immune surveillance, hepatocytes harboring genomic instability may gain a relative survival advantage, which over prolonged periods could facilitate progression toward hepatocellular carcinoma.

## PQC-antigen presentation crosstalk as a driver of HBV-related hepatocarcinogenesis

Conceptually, the pathways discussed in this review can be organized into three functional tiers. First, chronic viral protein production imposes primary proteotoxic stress, activating ER stress and UPR signaling. Second, compensatory adaptations in the UPS and autophagy reshape intracellular proteostasis and metabolic signaling. Third, sustained perturbation of these PQC systems compromises antigen processing and presentation, reducing immune surveillance. We suggest that the first tier represents the initiating pressure, the second tier reflects adaptive remodeling, and the third tier mediates immune escape and clonal persistence.

**FIGURE 2:**
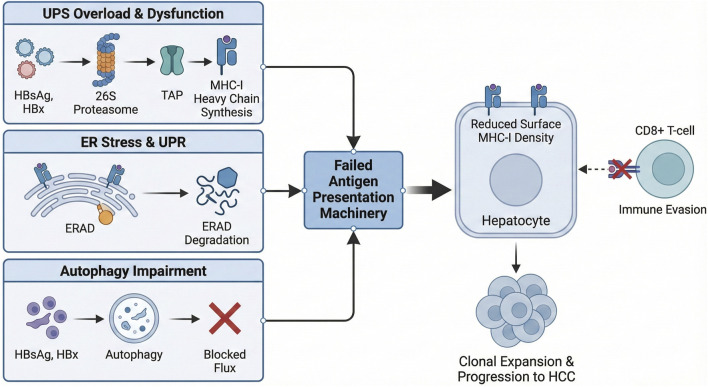
PQC–Antigen Presentation Crosstalk Driving Immune Escape. (Left) Chronic HBV infection induces massive proteotoxic stress. (Top) Viral protein overload saturates the ubiquitin-proteasome system (UPS) and suppresses immunoproteasome induction, reducing peptide generation. (Middle) In the ER, chronic UPR activation reduces the synthesis of MHC-I and TAP. Unloaded MHC-I molecules are unstable and targeted for degradation via ERAD. (Bottom) Blocked autophagic flux prevents the processing of alternative antigens. (Right) The cumulative result is a severe reduction in surface MHC-I density, rendering the hepatocyte “invisible” to CD8^+^ T-cells and permitting the clonal expansion of malignant cells.

Protein quality control (PQC) and antigen presentation are often treated as separate biological domains, yet in hepatocytes infected with HBV, they form an intimately connected system whose dysfunction is central to viral persistence and malignant evolution (Figure 2). HBV simultaneously increases folding and degradation demands, manipulates the UPS and autophagy, and suppresses the processing and presentation of viral antigens. The resulting collapse of PQC-immune homeostasis enables hepatocytes to survive in a chronically stressed environment while escaping immune surveillance (Wang et al., 2006; Tang et al., 2009).

At the level of proteasomal function, PQC disruption has a direct impact on antigen presentation. Proteasomal overload induced by the vast quantities of HBV proteins reduces the efficiency of cleavage events necessary for generating high-affinity viral peptides (Murphy et al., 2016; Chen et al., 2006). UPR signaling also plays a central role in linking PQC dysfunction to antigen presentation failure. PERK activation lowers global translation and decreases the supply of proteins available for antigen processing (Mazzolini et al., 2025). IRE1α-dependent mRNA decay targets transcripts encoding components of the antigen presentation machinery (Osorio et al., 2014). HBV-induced activation of ATF6 increases ER chaperone demand and contributes to global ER stress, diverting folding capacity toward viral proteins (Li et al., 2007). Under these conditions, MHC-I heavy chains are inefficiently folded and increasingly targeted to ERAD, a process that reduces peptide–MHC-I surface expression (Zhu et al., 2021). HBV further amplifies this effect by enhancing ERAD activity through HBx-mediated modulation of ER stress pathways (Cho et al., 2011).

Autophagy adds another dimension to this crosstalk. Under physiological conditions, autophagy supports antigen presentation by delivering cytoplasmic antigens for MHC-II processing and contributing to cross-presentation. But HBV-induced inhibition of autophagosome-lysosome fusion disrupts lysosomal degradation and decreases MHC-II peptide availability (Silva and Jung, 2013). Incomplete autophagy also increases the burden on the UPS, creating a proteostasis bottleneck that diminishes peptide quality for MHC-I loading (Li et al., 2022).

Collectively, these PQC defects ensure that hepatocytes present fewer viral epitopes, present them less efficiently, and provide fewer activation cues to cytotoxic T cells. This immunologically silent environment allows damaged hepatocytes-laden with oxidative lesions, genomic instability, and sublethal mutations-to escape immune clearance. As these cells divide and accumulate additional alterations, they advance along a trajectory toward hepatocellular carcinoma (Zheng et al., 2025).

Thus, PQC dysfunction is not merely a byproduct of viral replication but a mechanistic driver of immune escape and malignant transformation. The tight coupling of PQC and antigen presentation reveals a unifying concept: HBV-related hepatocarcinogenesis arises from the failure of the hepatocyte to maintain proteostasis and immunological visibility under chronic viral pressure.

## Clinical implications: biomarkers and therapeutic opportunities

The mechanistic integration of PQC disruption and antigen presentation failure in chronic HBV infection has important clinical implications for biomarker development and therapeutic innovation. Several PQC-associated markers show promise for identifying patients at elevated risk of hepatocellular carcinoma. ER stress indicators such as GRP78, CHOP, and spliced XBP1 are consistently elevated in chronically infected liver tissue and correlate with progressive disease (Olivares and Henkel, 2015; Ji et al., 2011; DeZwaan-McCabe et al., 2013). The presence of pre-S mutant-induced “ground-glass hepatocytes,” which reflect intracellular accumulation of misfolded HBs proteins, has emerged as a histological signature of precancerous lesions and may help stratify surveillance intensity (Pollicino et al., 1995). Dysregulation of UPS components also carries diagnostic potential; altered proteasome activity or reduced expression of immunoproteasome subunits, particularly LMP2 and LMP7, suggests impaired antiviral antigen processing (Ebstein et al., 2012). Similarly, autophagy markers such as persistent LC3-II accumulation or elevated p62/SQSTM1 signal incomplete autophagy flux typical of HBV-infected hepatocytes and may reflect underlying oxidative stress and genomic instability (Wang L. et al., 2019; Runwal et al., 2019; Liang et al., 2024). Decreased MHC-I surface expression provides another functional readout of compromised antigen presentation and may serve as an early indicator of immune escape preceding malignant evolution.

Therapeutic interventions targeting PQC pathways may offer opportunities both to ameliorate hepatocyte stress and to interfere with biological processes that contribute to HBV-related carcinogenesis. Modulating the UPR could, in principle, shift hepatocytes from maladaptive survival programs toward more balanced proteostasis or controlled apoptosis. Inhibitors of IRE1α RNase activity or PERK signaling have shown promise in preclinical cancer models and might reduce pro-tumorigenic stress adaptation in HBV-related disease, although their safety and efficacy in chronically injured liver remain to be established (Crowley et al., 2023; Zhang et al., 2024). Conversely, enhancing selected adaptive UPR branches could alleviate ER stress and limit oxidative damage, but the net impact on viral replication and tumor evolution is still uncertain (Cao and Kaufman, 2014). Manipulating proteasome function requires caution due to hepatotoxicity, yet selectively restoring or augmenting immunoproteasome activity may improve the generation of immunogenic viral peptides and thereby enhance antiviral immune recognition (Zhao et al., 2025; Kim et al., 2022). Autophagy modulation presents another potential avenue; agents that restore autophagosome–lysosome fusion may reduce mitochondrial dysfunction and ROS accumulation in experimental systems, while controlled autophagy inhibition has been reported to suppress HBV replication in some models, although careful calibration is necessary to avoid exacerbating proteotoxic burden or impairing hepatocyte resilience (Wei et al., 2025; Kim et al., 2024; Shyam et al., 2021).

Interventions aimed at improving antigen presentation represent a particularly compelling conceptual strategy to enhance antiviral and antitumor immunity. Interferon-based therapies, despite declining use, remain among the few clinically deployed treatments that can upregulate immunoproteasome subunits and TAP expression, thereby potentially increasing viral epitope presentation in hepatocytes (Bergman et al., 2011). Epigenetic regulators capable of restoring or augmenting MHC-I transcription may increase hepatocyte visibility to cytotoxic T cells, although this approach remains largely preclinical in HBV-related settings (Wijdeven et al., 2024; Dandri, 2020). Small molecules designed to disrupt the HBx–DDB1 interaction could, in principle, stabilize the SMC5/6 complex and limit viral transcription, indirectly easing antigen processing by reducing viral protein load, but such agents have yet to be fully developed or validated clinically (Allweiss et al., 2022). Therapies that mitigate ER stress, such as chemical chaperones, may also improve MHC-I maturation and trafficking in model systems, suggesting a possible route to enhance antigen presentation, though direct evidence in HBV-infected human livers is still limited (Hu et al., 2022; Lu et al., 2025).

These insights have important implications for immunotherapy in HBV-related HCC. Checkpoint inhibitors such as PD-1 and PD-L1 antibodies have shown modest efficacy overall, and the limited response rates likely reflect multiple mechanisms of immune evasion, with defects in antigen presentation representing one plausible contributing factor (Zhou et al., 2023; Su et al., 2024). Approaches aimed at increasing the abundance or quality of tumor-associated or viral antigens presented on hepatocytes, including PQC-targeted therapies, could sensitize tumors to checkpoint blockade (Chai et al., 2025; Shen et al., 2022). Therapeutic vaccines targeting conserved HBV epitopes might likewise become more effective once antigen presentation pathways are at least partially restored (Jansen et al., 2021). Together, these strategies point toward a potential therapeutic paradigm in which restoring proteostasis and antigen presentation complements, and may substantially enhance, traditional antiviral suppression in efforts to reduce the burden of HBV-related hepatocellular carcinoma.

## Temporal dynamics and cellular heterogeneity in chronic HBV infection

Chronic HBV infection unfolds over decades and is characterized by fluctuating viral loads, immune activity, and degrees of liver injury. PQC alterations and antigen presentation defects are unlikely to be static features throughout this continuum. During early immune-tolerant phases, high viral protein production may impose substantial proteotoxic stress with relatively limited inflammatory pressure (Bertoletti and Kennedy, 2015). In contrast, during immune-active phases, inflammatory cytokines may amplify ER stress while simultaneously promoting compensatory hepatocyte proliferation (Schmidt-Arras and Rose-John, 2016).

Moreover, hepatocyte responses are heterogeneous. Single-cell analyses suggest variability in UPR activation, mitochondrial integrity, and antigen presentation capacity among infected cells (Jin et al., 2024). It is conceivable that malignant clones preferentially arise from hepatocyte subsets in which proteostasis adaptation permits survival, but antigen presentation is sufficiently impaired to avoid immune elimination. Understanding this spatial and temporal heterogeneity will be essential for refining the proposed PQC–immune axis model.

## Open questions and future directions

Despite substantial advances in understanding how HBV dysregulates protein quality control and antigen presentation, several critical questions remain unresolved. A major gap concerns the temporal sequence of PQC disruption during chronic infection: it is unclear whether ER stress, autophagy defects, and UPS overload arise early as primary pathogenic events or develop secondarily as hepatocytes adapt to persistent viral antigen burden. Likewise, the identification of PQC components most predictive of malignant transformation remains an open challenge, underscoring the need for longitudinal studies integrating ER stress signatures, autophagy flux markers, immunoproteasome activity, and antigen presentation profiles to refine HCC risk stratification. HBV itself is heterogeneous, and different genotypes or mutations-particularly pre-S deletions and core promoter variants-may impose distinct proteostatic stresses or immune-evasion strategies, suggesting that viral genetic diversity could shape the trajectory toward carcinogenesis. Another emerging issue is cellular heterogeneity within HBV-infected livers: hepatocytes differ markedly in UPR activation, mitochondrial quality control, antigen presentation capacity, and susceptibility to DNA damage, raising the possibility that malignant clones arise from specific spatial or molecular niches. Advanced techniques such as single-cell RNA sequencing, spatial transcriptomics, and deep proteomics will be essential for unraveling this complexity. Finally, although PQC pathways represent promising therapeutic targets, the challenge lies in safely modulating these systems in a chronically injured liver that depends heavily on robust proteostasis for metabolic and detoxification functions. Developing treatments that selectively correct maladaptive PQC responses without impairing physiological proteostasis will be key to translating mechanistic insights into clinical interventions for HBV-related HCC.

## Conclusion

Rather than representing isolated pathways, protein quality control and antigen presentation form an integrated stress–immunity interface in chronically infected hepatocytes. We propose that persistent viral proteotoxic stress initiates adaptive PQC remodeling, which in turn progressively erodes antigen presentation capacity. This dual adaptation permits hepatocytes to survive intracellular stress while simultaneously reducing immunological visibility. Over prolonged infection, this environment favors clonal persistence of genomically unstable cells and increases the probability of malignant transformation.

The convergence of PQC dysfunction and antigen presentation impairment forms a mechanistic axis through which HBV establishes chronic persistence and drives hepatocarcinogenesis. This framework provides new opportunities for biomarker development, risk stratification, and therapeutic innovation. Ultimately, strategies that restore proteostasis and enhance antigen presentation, combined with antiviral or immunotherapeutic approaches, hold promise for reducing the global burden of HBV-related HCC.
